# The Importance of Large-Diameter Trees to Forest Structural Heterogeneity

**DOI:** 10.1371/journal.pone.0082784

**Published:** 2013-12-20

**Authors:** James A. Lutz, Andrew J. Larson, James A. Freund, Mark E. Swanson, Kenneth J. Bible

**Affiliations:** 1 Wildland Resources Department, Utah State University, Logan, Utah, United States of America; 2 Department of Forest Management, University of Montana, Missoula, Montana, United States of America; 3 School of Environmental and Forest Sciences, University of Washington, Seattle, Washington, United States of America; 4 School of the Environment, Washington State University, Pullman, Washington, United States of America; The Pennsylvania State University, United States of America

## Abstract

Large-diameter trees dominate the structure, dynamics and function of many temperate and tropical forests. However, their attendant contributions to forest heterogeneity are rarely addressed. We established the Wind River Forest Dynamics Plot, a 25.6 ha permanent plot within which we tagged and mapped all 30,973 woody stems ≥1 cm dbh, all 1,966 snags ≥10 cm dbh, and all shrub patches ≥2 m^2^. Basal area of the 26 woody species was 62.18 m^2^/ha, of which 61.60 m^2^/ha was trees and 0.58 m^2^/ha was tall shrubs. Large-diameter trees (≥100 cm dbh) comprised 1.5% of stems, 31.8% of basal area, and 17.6% of the heterogeneity of basal area, with basal area dominated by *Tsuga heterophylla* and *Pseudotsuga menziesii*. Small-diameter subpopulations of *Pseudotsuga menziesii*, *Tsuga heterophylla* and *Thuja plicata*, as well as all tree species combined, exhibited significant aggregation relative to the null model of complete spatial randomness (CSR) up to 9 m (*P*≤0.001). Patterns of large-diameter trees were either not different from CSR (*Tsuga heterophylla*), or exhibited slight aggregation (*Pseudotsuga menziesii* and *Thuja plicata*). Significant spatial repulsion between large-diameter and small-diameter *Tsuga heterophylla* suggests that large-diameter *Tsuga heterophylla* function as organizers of tree demography over decadal timescales through competitive interactions. Comparison among two forest dynamics plots suggests that forest structural diversity responds to intermediate-scale environmental heterogeneity and disturbances, similar to hypotheses about patterns of species richness, and richness- ecosystem function. Large mapped plots with detailed within-plot environmental spatial covariates will be required to test these hypotheses.

## Introduction

Large, persistent woody structures (large-diameter trees, snags, and logs) and spatial heterogeneity are defining characteristics of late-successional forests [1], [2]. Large-diameter trees (here defined as those with a diameter ≥100 cm at breast height (1.37 m; dbh) contribute disproportionately to ecosystem function [3], [4], including biomass and carbon storage [5], [6]. The heterogeneous structure of late-successional forests includes variation in tree density and size across the landscape [7], [8], [9], as well as the variation in vertical canopy structure [10], [11] and tree crown architecture [12], [13].

The relative rarity and low demographic rates (mortality and recruitment into large diameter classes) of large-diameter trees frequently render their investigation intractable [14], and, therefore, despite their exceptional ecological and social importance, large tree subpopulations remain relatively unstudied. In previous work in a late-successional mixed-conifer forest [6] we found that predictions for large-diameter tree abundance and spatial patterns based on scaling theory and competition theory did not agree with empirical observations. We also found that the largest 1.4% of trees accounted for 49.4% of aboveground biomass [6], underscoring the importance of large trees for providing the ecosystem service of carbon storage. This earlier work, however, was based on a single study site, the Yosemite Forest Dynamics Plot (YFDP). Our conclusions about the unique contribution of large-diameter trees to forest structure and function would be much stronger, and more generalizable, if replicated elsewhere.

Understanding drivers of spatial heterogeneity of aboveground biomass in forests is of considerable basic and applied ecological interest [15], [16]. Although biomass best represents many elements of ecosystem function, allometric equations for large-diameter trees embody considerable uncertainty [6]. Basal area is a measured quantity, and therefore more precise for comparisons at hectare scales. While spatial variation of aboveground biomass (or basal area) is obvious in many late-successional forests (e.g., [17], [18]), the degree to which large-diameter trees induce this spatial heterogeneity remains unknown.

Intermediate-scale (here defined as 100 m^2^ to 6400 m^2^) spatial variability of basal area should depend largely on the spatial arrangement of individual large-diameter trees. If large-diameter tree locations are aggregated due to, for example, habitat associations, dispersal limitations [19], or the spatial pattern that results from a particular disturbance regime (disturbance refugia) [20] spatial heterogeneity of forest basal area would be greater than if large-diameter trees are distributed in a spatially random pattern. Conversely, if large trees are overdispersed, for example as an outcome of strong density-dependent mortality (either competitive mortality or Janzen-Connell-type effects) in earlier stages of forest succession [21], [22], then spatial heterogeneity of basal area should be less that obtained from aggregated or randomly distributed large trees.

This study was motivated by three purposes: (1) validate our earlier conclusions about the importance of large-diameter trees to ecosystem structure and function [6] with an independent data set from a different forest type, species complement, and environmental setting; (2) investigate sensitivity of intermediate-scale heterogeneity of basal area to the spatial distribution of large-diameter trees; and (3) support management efforts to restore the structure of previously-harvested late-successional forests [23]. We established the Wind River Forest Dynamics Plot (WFDP), a 25.6 ha permanent forest research plot, and within the plot quantified the relative contribution of large-diameter trees and snags to forest composition, structure, the comparative spatial patterns of large-diameter and small-diameter trees, and spatial relationships between them. We then investigated the sensitivity of spatial heterogeneity of basal area to the spatial distribution of large-diameter trees in both the WFDP and the paired YFDP with a simulation experiment based on spatial point process modeling.

## Results

### Species Composition

**Figure 1:**
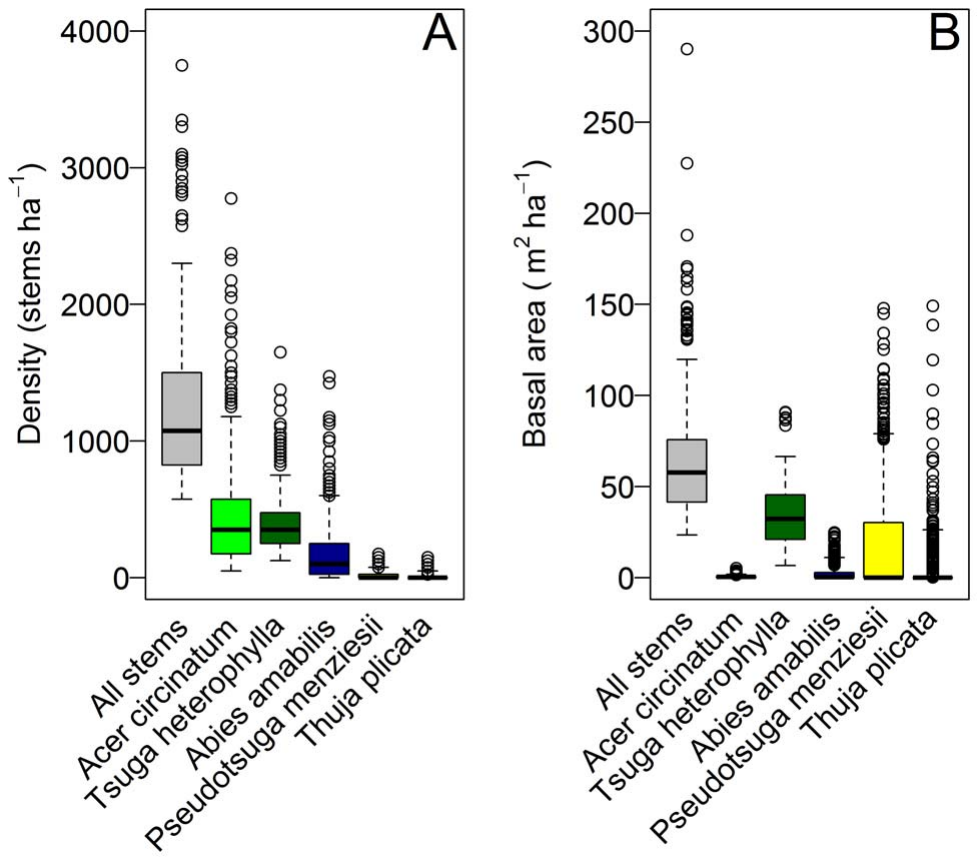
Heterogeneity in density and basal area of the five principal tree species of the Wind River Forest Dynamics Plot in 2012. Each boxplot represents values from the 640, 20×20 m quadrats of the plot. Boxplot boxes indicate the 25^th^ percentile, median, and 75^th^ percentile. Boxplot whiskers indicate the 5^th^ percentile and the 95^th^ percentile.

**Figure 2:**
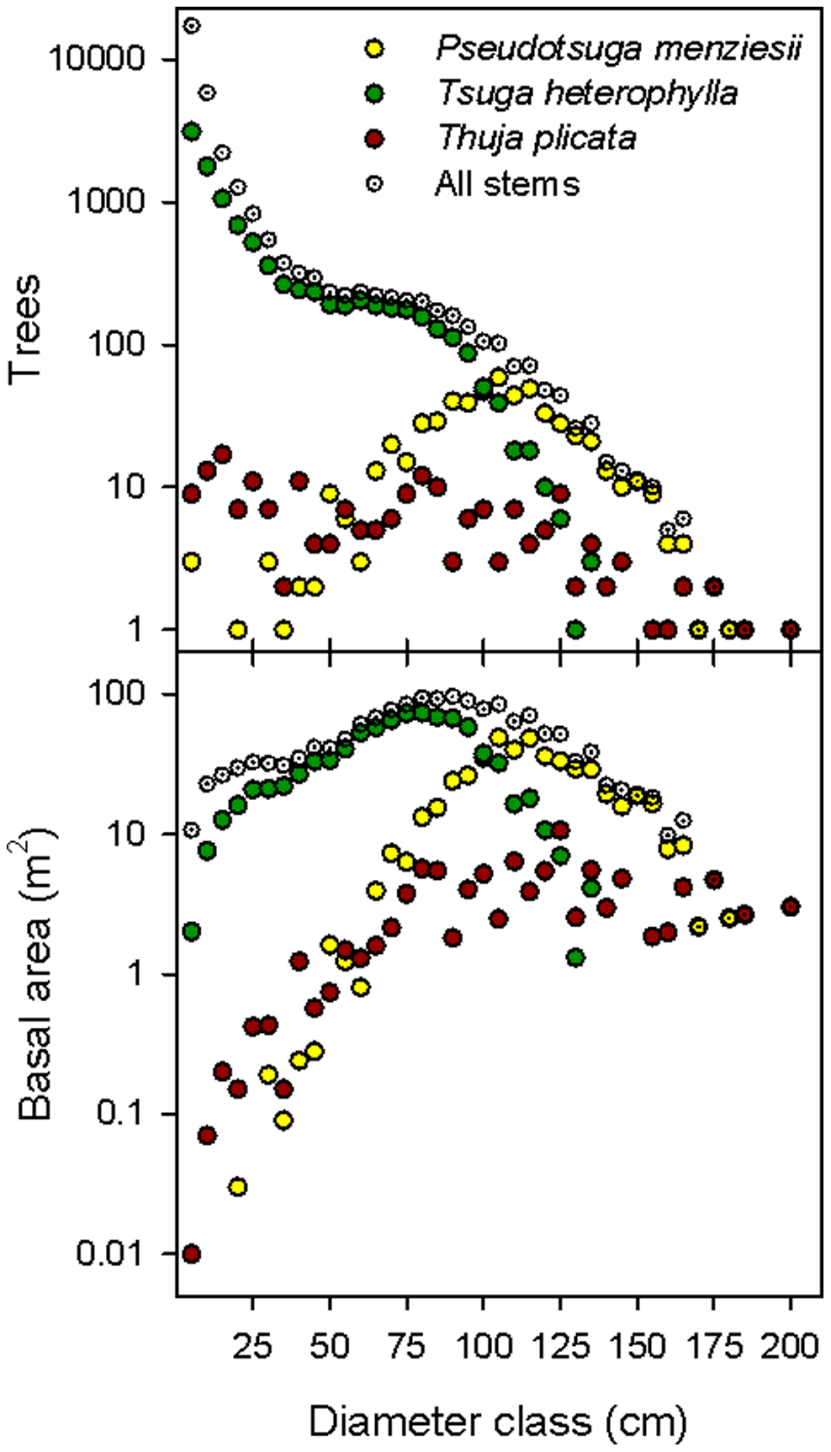
Diameter distribution of the number of trees and basal area for the three most common species in the Wind River Forest Dynamics Plot in 2012. Each point represents a 5(first bin; 1 cm≤dbh<5 cm) of the trees from the entire 25.6 ha plot (30,973 live stems ≥1 cm dbh totaling 62.18 m^2^/ha).

In the 25.6 ha of the WFDP, there were 30,973 live stems ≥1 cm dbh of 26 tree, shrub, and liana species (Table 1) and 2.4 ha (9.4%) of continuous shrub and fern cover comprising species that rarely reach the 1 cm dbh threshold (Table 2). Nine woody plant families were represented. All woody stems were native plants. Live tree and shrub basal area ≥1 cm dbh was 61.60 m^2^/ha. The three principal species by basal area (*Pseudotsuga menziesii*, *Tsuga heterophylla*, and *Thuja plicata*) constituted 34.6% of stems and 91.8% of basal area, but were highly variable at 20 m scales (Fig. 1). The most abundant species by number of stems, *A. circinatum*, comprising 35.8% of all stems, was aggregated near vernal watercourses, but occurred in 97.8% of 20 m×20 m quadrats. *Tsuga heterophylla* was slightly less abundant (32.0% of stems), but was more uniformly distributed, occurring in all 640 quadrats. *Abies amabilis* comprised 14.4% of all stems but was fully 37.5% of all trees <10 cm dbh. Diameter distributions of *P. menziesii* exhibited a bell shaped distribution, relatively symmetric and unimodal, consistent with its life history as a shade-intolerant pioneer (Fig. 2). The diameter distribution of *T. plicata* followed a negative exponential distribution, and the diameter distribution of *T. heterophylla* followed a rotated sigmoid distribution (Fig. 2). There were 1,966 snags ≥10 cm dbh (Table 1).

**Table 1:** Woody plant species within the Wind River Forest Dynamics Plot in 2012.

Tree species	Family	Density(stems/ha)	Stems ≥1 cmdbh	Stems ≥10 cm dbh	Stems ≥100 cm dbh	Large-diameter prop. (%)	Basal area ≥1 cm(m^2^/ha)	Basal area ≥10 cm(m^2^/ha)	Basal area ≥100 cm(m^2^/ha)	Large-diameter prop. (%)
Trees ≥1 cm										
* Tsuga heterophylla*	Pinaceae	387.9	9,929	5,080	93	0.9%	34.09	33.72	3.41	10.0%
* Abies amabilis*	Pinaceae	173.1	4,431	984	1	0.0%	2.24	2.00	0.03	1.5%
* Taxus brevifolia*	Taxaceae	79.7	2,041	1,326	–	–	1.68	1.56	0.00	0.0%
* Pseudotsuga menziesii*	Pinaceae	22.3	572	569	308	53.8%	19.14	19.14	13.82	72.2%
* Thuja plicata*	Cupressaceae	7.9	201	180	47	23.4%	3.88	3.88	2.46	63.5%
* Cornus nuttallii*	Cornaceae	6.7	172	41	–	–	0.04	0.02	0.00	0.0%
* Abies grandis*	Pinaceae	2.0	51	36	–	–	0.24	0.24	0.00	0.0%
* Alnus rubra*	Betulaceae	0.6	16	4	–	–	0.01	0.01	0.00	0.0%
* Abies procera*	Pinaceae	0.5	12	11	–	–	0.17	0.17	0.00	0.0%
* Pinus monticola*	Pinaceae	0.2	6	6	2	33.3%	0.13	0.13	0.08	62.7%
Live tree total		680.9	17,431	8,237	451	2.6%	61.60	60.86	19.80	32.1%
Tall shrubs ≥1 cm										
* Acer circinatum*	Sapindaceae	432.9	11,081	90	–	–	0.56	0.04	–	–
* Vaccinium parvifolium*	Ericaceae	48.8	1,250	–	–	–	0.01	–	–	–
* Corylus cornuta* var. *californica*	Betulaceae	24.5	628	–	–	–	0.01	–	–	–
* Rhododendron macrophyllum*	Ericaceae	17.9	458	–	–	–	0.01	–	–	–
* Vaccinium ovalifolium*	Ericaceae	2.3	59	–	–	–	t	–	–	–
* Holodiscus discolor*	Rosaceae	0.7	19	–	–	–	t	–	–	–
* Menziesia ferruginea*	Ericaceae	0.7	17	–	–	–	t	–	–	–
* Gaultheria shallon*	Ericaceae	0.5	13	–	–	–	t	–	–	–
* Amelanchier alnifolia*	Rosaceae	0.3	8	–	–	–	t	–	–	–
* Oemleria cerasiformis*	Rosaceae	0.1	3	–	–	–	t	–	–	–
* Acer glabrum*	Sapindaceae	t	1	–	–	–	t	–	–	–
* Rosa gymnocarpa*	Rosaceae	t	1	–	–	–	t	–	–	–
* Rubus leucodermis* †	Rosaceae	t	1	–	–	–	t	–	–	–
* Rubus spectabalis*	Rosaceae	t	1	–	–	–	t	–	–	–
* Vaccinium membranaceum*	Ericaceae	t	1	–	–	–	t	–	–	–
Tall shrub total		528.8	13,541	90	–	–	0.58	0.04	–	–
Liana ≥1 cm										
* Lonicera ciliosa*	Caprifoliaceae	t	1	–	–	–	–	–	–	–
Live woody stem total		1,209.7	30,973	8,327	451	1.5%	62.18	60.90	19.80	31.8%
Snags ≥10 cm										
* Pseudotsuga menziesii*				786	118	15.0%		14.34	5.10	35.6%
* Tsuga heterophylla*				399	18	4.5%		3.89	0.70	18.0%
* Taxus brevifolia*				302	–	–		0.27	–	–
* Abies amabilis*				175	–	–		0.70	–	–
* Pinus monticola*				62	6	9.7%		1.08	0.22	20.5%
* Abies grandis*				31	–	–		0.23	–	–
* Thuja plicata*				27	1	3.7%		0.16	0.03	22.0%
* Acer circinatum*				7	–	–		0.00	–	–
* Cornus nuttallii*				3	–	–		0.00	–	–
* Abies* spp.				2	–	–		0.01	–	–
* Abies procera*				1	1	100.0%		0.03	0.03	100.0%
* Alnus rubra*				1	–	–		0.00	–	–
Unknown				170	5	2.9%		1.66	0.20	12.0%
Dead tree total				1,966	149	7.6%		22.37	6.29	28.1%

t – trace; density less than one tree per 10 ha; basal area less than 0.01 m^2^/ha.

† Biennial canes.

**Table 2:** Low shrub and fern species occurring in patches of continuous cover ≥2 m^2^ in the Wind River Forest Dynamics Plot in 2012.

Species	Family	Cover (m^2^)
*Athyrium filix-femina*	Dryopteridaceae	2.4
*Blechnum spicant*	Blechnaceae	1,340.1
*Gaultheria shallon* †	Ericaceae	8,903.5
*Mahonia nervosa*	Berberidaceae	10,331.3
*Menziesia ferruginea* ‡	Ericaceae	2.7
*Polystichum munitum*	Dryopteridaceae	56.2
*Pteridium aquilinum*	Dennstaedtiaceae	298.3
*Rhododendron macrophyllum* ‡	Ericaceae	899.0
*Rubus spectabalis*	Rosaceae	8.7
*Thelypteris nevadensis*	Thelypteridaceae	1,884.9
*Vaccinium ovalifolium* ‡	Ericaceae	70.8
*Vaccinium parvifolium* ‡	Ericaceae	239.8
Total		24,037.7

† Principally of low stature, but occasionally reaches heights and diameters sufficient to enter the population of tagged stems.

‡ Plants that usually attain statures sufficient to enter the population of tagged stems. Areas listed represent continuous cover of plants not large enough to be tagged.

### Large-diameter Tree Composition

The large diameter component (≥100 cm dbh) constituted 1.5% of stems and 32.1% of basal area. However, some species were represented almost exclusively in the large diameter class. *Pseudotsuga menziesii*, the shade-intolerant early seral species had 53.8% of stems and 72.2% of basal area in diameters ≥100 cm dbh. *T. plicata*, with 23.4% of stems and 63.5% of basal area concentrated in the ≥100 cm dbh diameter class, included the largest diameter trees, 184.2 cm dbh and 196.2 cm dbh, potentially reflecting individuals that survived the stand initiating disturbance. The diameter structure of *Pinus monticola*, with 33.3% of stems and 62.7% of basal area concentrated in the ≥100 cm dbh diameter class, represents both its shade-intolerant, early seral life-history, and also the effects of *Cronartium ribicola* (white pine blister rust), which has caused higher mortality and lack of regeneration since the 1930s. Indeed, standing snags of *P. monticola* outnumber living individuals by a factor of ten. The coefficient of variation of basal area at the scale of the 20 m×20 m quadrats was 0.51 for all trees, but only 0.42 for trees <100 cm dbh, which is to say that although only 1.5% of the tree population, large-diameter trees accounted for 17.6% of the variation in basal area.

### Spatial Patterns

**Figure 3:**
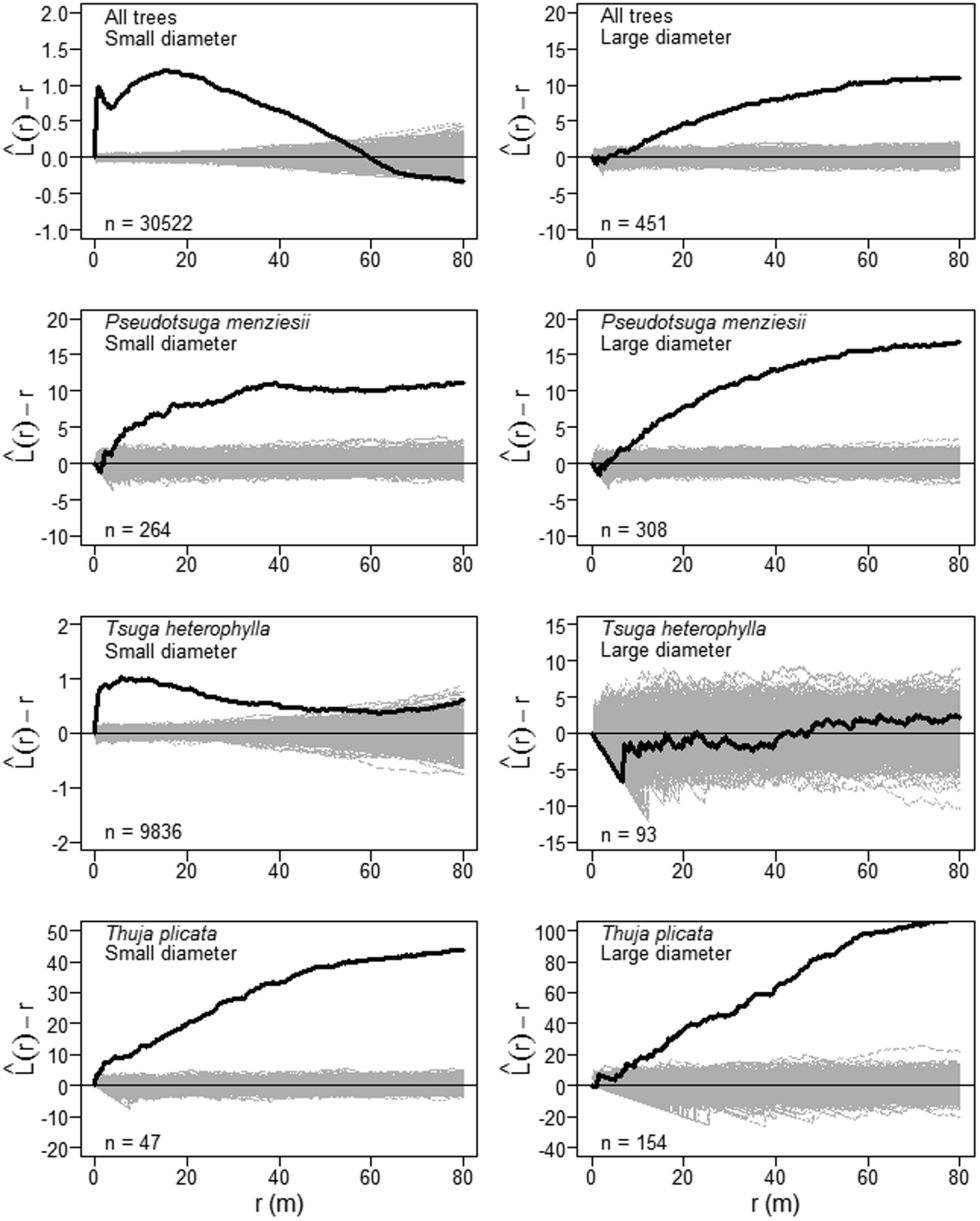
Univariate spatial patterns of tree species that attain large diameters in the Wind River Forest Dynamics Plot. Solid black lines show the 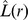 statistic for the actual patterns, where *r* is the intertree distance; thin gray lines show 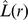 curves for 999 simulations of complete spatial randomness. Positive values of 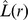 indicate spatial clumping and negative values of 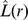 indicate spatial regularity. Large-diameter trees are ≥100 cm dbh; small-diameter trees are <100 cm dbh.

Small-diameter subpopulations (1 cm≤dbh<100 cm) of *P. menziesii*, *T. heterophylla* and *T. plicata*, as well as all tree species combined, exhibited significant aggregation relative to the null model of complete spatial randomness (CSR) up to 9 m (Monte Carlo goodness-of-fit test; *P. menziesii*: *P* = 0.001; *T. heterophylla*: *P* = 0.001; *T. plicata*: *P* = 0.001; all trees: *P* = 0.001). In other words, when averaged across all points in a given pattern, small-diameter trees of these species have more neighbors of the same type located within a circle with a radius of 9 m that would be expected if tree locations were completely independent of each other. 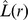 values (the square root transformation of the Ripley’s *K* function – see Materials and Methods) for small-diameter stems of *T. heterophylla* and *T. plicata* rose steeply at the smallest small scales (<2 m), while those of *P. menziesii* were spatially random over the first few meters then becoming statistically aggregated beyond about 4 m (Fig. 3 and Fig. S1).

The spatial arrangement of large-diameter *T. heterophylla*, as well as all tree species combined, was not statistically different from spatial randomness from 0 m to 9 m (Monte Carlo goodness-of-fit test; *T. heterophylla*: *P* = 0.211; all trees: *P* = 0.133). However, visual inspection of the empirical 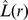 values for large-diameter *T. heterophylla* over these same distances (Figs. 3 and S1) suggests spatial inhibition which might be confirmed by a larger sample size. Post-hoc power analysis (data not shown) indicated that a hard core spatial inhibition process could not be differentiated from complete spatial randomness at the observed intensity of large-diameter *T. heterophylla*. Thus, while there was some evidence for ecologically significant spatial regularity in the locations of *T. heterophylla* up to about 6.5 m (middle right panel of Fig. S1), these large-diameter individuals were not abundant enough for us to detect a statistically significant difference from CSR. In contrast, large-diameter individuals of *P. menziesii* and *T. plicata* were slightly spatially aggregated at distances up to 9 m (Monte Carlo goodness-of-fit test; *P. menziesii*: *P* = 0.003; *T. plicata*: *P* = 0.005). However, in both cases the empirical 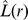 values indicated spatial inhibition at very small scales (<1.5 m)–reflecting the physical requirement for a minimum intertree spacing for these large trees–then transitioning to spatial aggregation at scales larger than about 2 m (Fig. S1).

**Figure 4:**
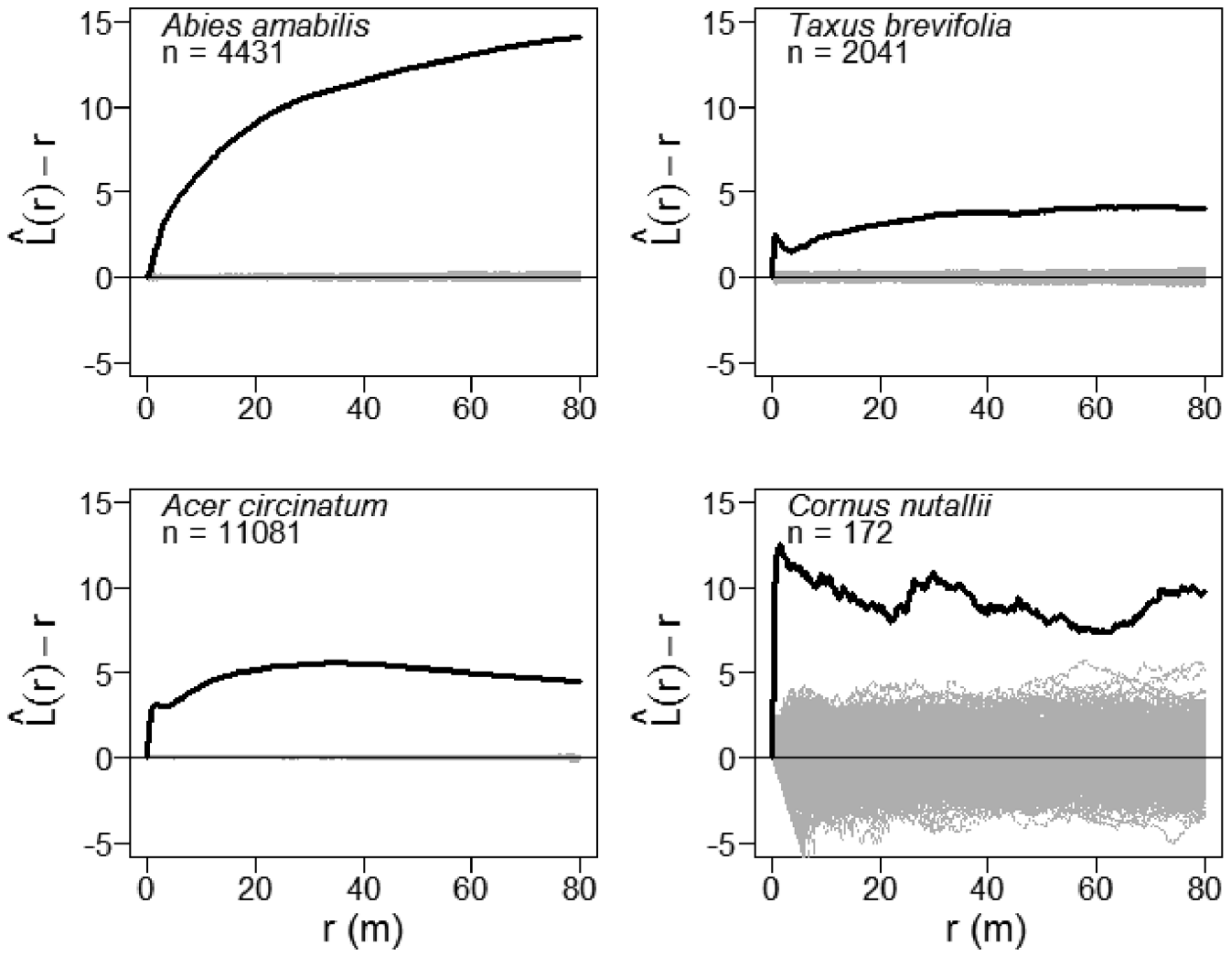
Univariate spatial patterns for abundant small-diameter tree species in the Wind River Forest Dynamics Plot. Solid black lines show the 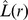 statistic for the actual patterns, where *r* is the intertree distance; thin gray lines show 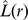 curves for 999 simulations of complete spatial randomness. Positive values of 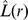 indicate spatial clumping and negative values of 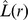 indicate spatial regularity.

Exploratory analysis of the three most abundant small-diameter tree species, *Abies amabilis*, *Taxus brevifolia*, and *Cornus nuttallii*, and the most abundant tall shrub species, *Acer circinatum* (Table 1), revealed strong spatial aggregation at scales <1.5 m (Figs. 4 and S2). *Abies amabilis* continued to exhibit spatial aggregation at larger scales: i.e., the 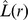 value continued to increase (Fig. 4). In contrast, the 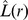 values for *T. brevifolia*, *A. circinatum*, and *C. nuttallii* all plateaued at scales beyond about 1.5 m to 2.0 m (Fig. 4), indicating that the processes driving the spatial aggregation of these species operate at only the smallest intertree distances.

**Figure 5:**
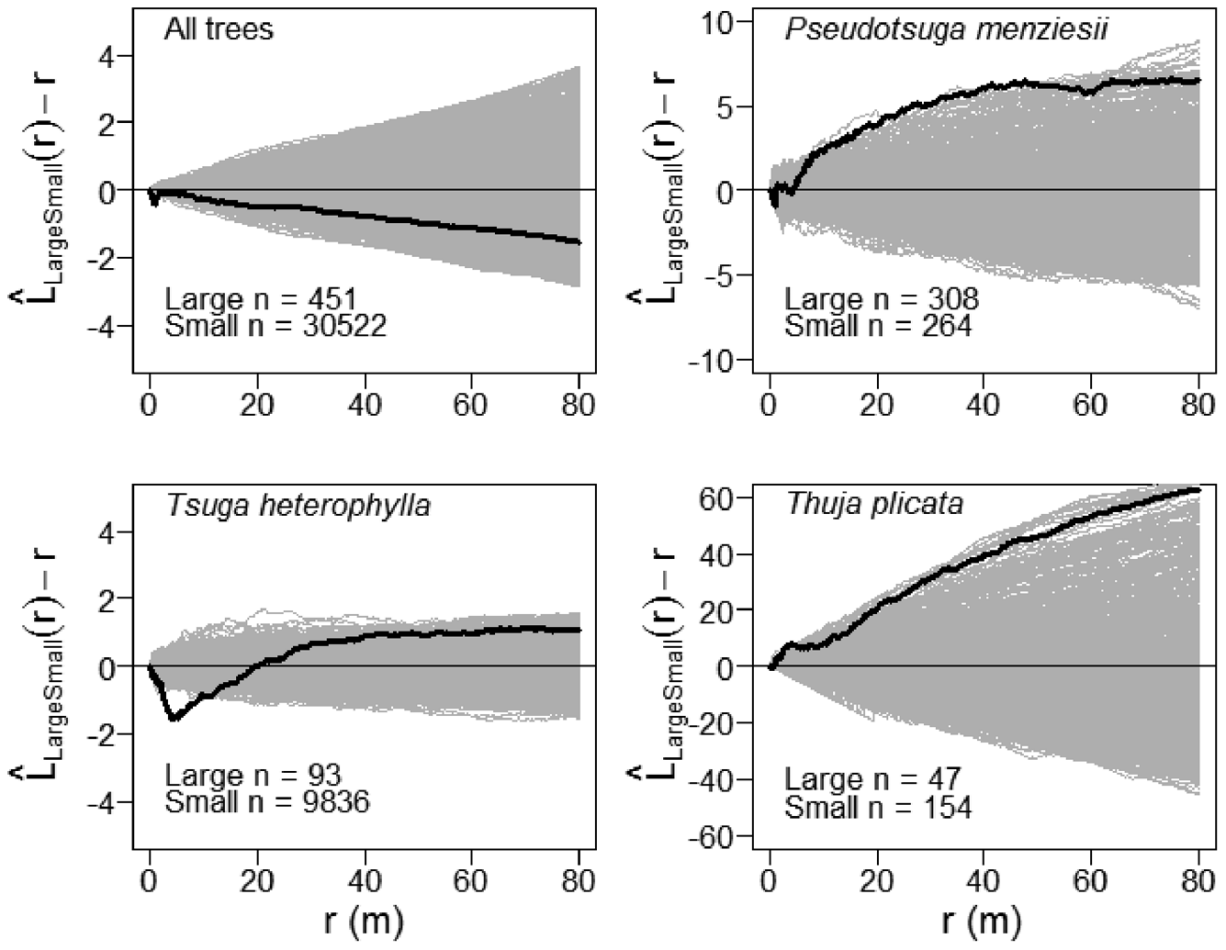
Spatial interactions between large-diameter and small-diameter trees. Solid black lines show the 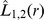 statistic for the actual pattern, where *r* is the intertree distance; thin gray lines show 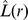 curves for 999 patterns simulated by synchronous random torodial shifts of large- and small-diameter tree subpopulations. Positive values of 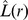 indicate spatial attraction and negative values of 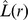 indicate spatial repulsion. Large-diameter trees are ≥100 cm dbh; small-diameter trees are <100 cm dbh.

We found strong spatial repulsion between large-diameter and small-diameter *T. heterophylla* (Monte Carlo goodness-of-fit test; *P* = 0.001). The empirical 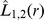 (Figs. 5 and S3) indicate that this spatial repulsion peaks at about 5 m to 6 m. In contrast, there was modest evidence for spatial attraction between conspecific small- and large-diameter *P. menziesii*, and *T. plicata*, respectively (Monte Carlo goodness-of-fit test; *P. menziesii*: *P* = 0.029; *T. plicata*: *P* = 0.008) at intertree distances up to 9 m (Figs. 5 and S3). The spatial attraction between size classes manifests between about 4 m to 10 m for *P. menziesii*, and from about 1 m to 4 m for *T. plicata* (Fig. S3). For all species combined there was no statistically significant difference from the null model of population independence at intertree distances up to 9 m (Monte Carlo goodness-of-fit test; *P* = 0.222). That said, for all species combined and for *P. menziesii* and *T. plicata*, inspection of the empirical 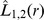 values showed strong repulsion between large- and small-diameter trees at scales up to about 1.5 m (Fig. S3).

### Spatial Heterogeneity of Basal Area

**Figure 6:**
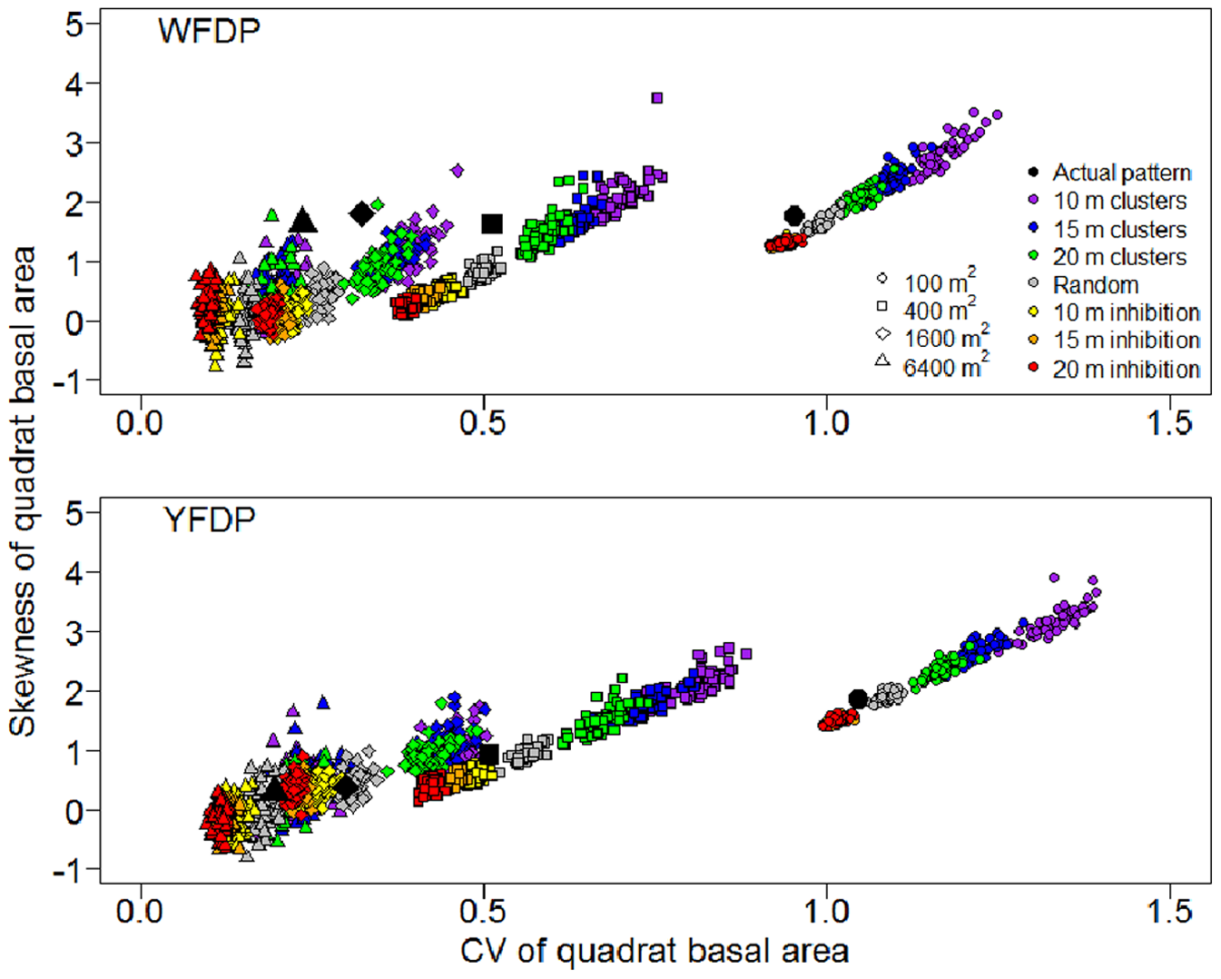
Dependence of aboveground basal area heterogeneity on spatial pattern of large-diameter trees in the Wind River Forest Dynamics Plot (WFDP) and the Yosemite Forest Dynamics Plot (YFDP). Large black symbols represent the actual patterns. Small symbols represent simulations.

The simulation experiment showed that heterogeneity of forest structure, as quantified by the coefficient of variation (CV) and skewness of quadrat basal area, was highly sensitive to both quadrat scale and spatial pattern of large-diameter trees at both the YFDP and WFDP. Both the CV and skewness of quadrat basal area increased with decreasing quadrat size (Fig. 6), as expected given that larger individual quadrats capture greater intra-quadrat variation of forest structure, thereby reducing inter-quadrat variation.

Spatial pattern of large-diameter trees strongly influenced both CV and skewness of quadrat basal area (Fig. 6). These metrics both increased along the pattern gradient from strong inhibition (i.e., uniformity), to spatial randomness, to clustering (i.e., aggregation). In other words, quadrat scale heterogeneity of forest structure (in basal area CV-skewness space) is highest when large-diameter trees are distributed in a globally aggregated pattern, and lowest when large-diameter trees are distributed in a globally uniform pattern (see Fig. S4 for examples of different pattern types). This general trend was invariant across quadrat scales, but the effect of pattern was maximized–that is, the pattern types were most strongly differentiated–at a quadrat scale of 400 m^2^ (20 m×20 m quadrats).

The empirical results (i.e., CV and skewness of quadrat basal area based on actual large-diameter tree locations) for the different quadrat sizes closely tracked those of CSR simulations for the YFDP (Fig. 6). The empirical results for the WFDP differed in an important way, however. Both CV and skewness were higher than expected at the larger three quadrat sizes, and this departure from the CSR expectation became more pronounced at the largest quadrat size. In other words, there is heterogeneity in the WFDP forest structure that is not explained by the second order spatial properties of the large-diameter tree locations.

## Discussion

Overall composition and structure of the WFDP is representative of older forests of the western Cascades [24], [25]. Although usually classified as a shrub and not considered in discussions of forest composition or structure, *A. circinatum* dominated the angiosperm component, and although comprising only 0.9% of the basal area, it was the most abundant woody species in terms of stem count. This is important because *A. circinatum* makes a disproportionate contribution to biodiversity in this evergreen conifer forest, for example by providing food for folivore geometrid larvae that feed Neotropical migrant birds [26] and by providing substrate for epiphytic lichens and bryophytes [27].

We found evidence for the subpopulation of large-diameter *T. heterophylla* acting as strong competitors within the forest community of the WFDP. Spatial repulsion between large and small *T. heterophylla* (Fig. 5) is evidence for strong intraspecific competition these two subpopulations. Additionally, the subpopulation of large-diameter *T. heterophylla* appears overdispersed up to about 6.5 m (Fig. S1). These results cause us to predict that large-diameter *T. heterophylla* will function as strong organizers of spatially-structured tree demography over decadal timescales through competitive interactions. Further, we anticipate that the outcomes of competition will differ qualitatively from that in young and mature single-cohort forests (e.g., [21]) developing through a typical structural development sequence following stand replacement disturbance (*sensu* [1]). Here, we expect recruitment of seedlings into small tree diameters (>1 cm dbh) will be strongly suppressed within the neighborhoods of large *T. heterophylla* [28]. This is because *T. heterophylla*, being very a shade tolerant tree with typically deep crown and high leaf area, will effectively preempt resources required for even stress-tolerating late successional tree species [29], [30] to grow into the small tree diameter class.

Notwithstanding the preceding discussion of large-diameter *T. heterophylla* functioning as strong competitors, we infer an overall limited role of past competitive mortality as a driver of tree spatial patterns when considering all species and size classes. Large-diameter trees of individual species and for all species pooled only show evidence of competition at very small scales (<1.5 m) as revealed by the 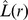 curves in Figure S1. This most likely reflects a minimum requirement for physical space (i.e. non-overlapping tree boles) and limits to crown plasticity. Earlier work within a 4 ha subregion of the WFDP did not support a strong role of competition at intertree distances up to 13 m [31]. Considering this earlier work [31] alongside Das et al.’s [32] finding of a limited role of competitive mortality in old-growth mixed conifer forests in the Sierra Nevada, and the congruent results obtained at the WFDP (this study) and the YFDP [6], we suggest a generally limited role for competitive mortality as an overall driver of tree spatial patterns in old-growth conifer forests. Thus, while we acknowledge a role for competitive interactions, particularly with large-diameter *T. heterophylla*, we must consider other factors such as small scale disturbance [14] and biotic tree mortality agents [33], [34] to fully explain the structure, pattern, and dynamics of old-growth conifer forests [1], [6].

Although our results suggest a limited role of competition, interpretation of mechanism based solely on one snapshot of static pattern contains many pitfalls. Other mechanisms besides intraspecific competition (i.e., temporal variation in seed production, patchy soil properties, presence or absence of seed or seedling pathogens, or mycorrhizal fungi) could cause the patterns we observed for large-diameter versus small-diameter *T. heterophylla*. In addition, the comparison of the sub-populations of large-diameter and small-diameter trees is not completely consistent among species, particularly in the case of *Pseudotsuga*, which has a diameter distribution skewed to larger diameters (Fig. 2). Collection of additional longitudinal data will improve the quality of the inferences (e.g. [35]).

We found that the most abundant tree species and tall shrubs of lesser physical stature (i.e., that do not reach 100 cm dbh) were consistently spatially aggregated, particularly at small spatial scales (Figs. 4 and S2). These patterns likely arise from a few key factors. In the case of *T. brevifolia* and *A. circinatum* this result is most likely explained, to a large degree, by the often multi-stemmed growth form of individual genets. Environmental heterogeneity and preferential recruitment, survival, and growth in canopy gaps are other likely drivers of this pattern, particularly for *A. amabilis* [36]. Our observational results are consistent with the response of *A. amabilis* seedlings and saplings to experimental canopy gap creation [37].

The apparent aggregation of large-diameter trees at scales >9 m detected in the exploratory analysis of tree patterns (Fig. 3) requires cautious interpretation. At the scale of the entire WFDP, large-diameter trees locations are not homogeneous: there is a gradient in large tree density from west to east (.). Thus, the apparent aggregation of large-diameter trees is most conservatively interpreted as the “virtual aggregation” described by Wiegand and Moloney [38]. Based on this result, we suggest that identifying factors that induce variation in the first-order intensity of large-diameter trees, such as environmental or resource gradients, will be essential to explaining development of spatial heterogeneity in old-growth forests.

Our simulation experiment demonstrated the sensitivity of forest structural heterogeneity, as measured by the CV and skewness of quadrat basal area, to the second order spatial characteristics of large-diameter trees (Fig. 6). That a forest structural property so strongly depends on just the locations of the largest 1% to 2% of trees confirms and extends our earlier conclusions about the ecological importance of this elite class of trees [6]. The clear implication of this result is the need to understand in detail the ecological processes that influence and regulate the spatial distribution of large-diameter trees – an aspect of forest ecology often overlooked because it requires large, mapped research plots (but see [6], [39]. Spatial patterns of large-diameter tree recruitment and mortality, and the ecological and environmental drivers thereof, are obvious priorities for future empirical research in old-growth forests.

Variation in the first order intensity (i.e., density) of large-diameter trees throughout the forest [40] appears to be an important determinant of basal area heterogeneity. This interpretation follows from the persistently elevated skewness of the empirical quadrat basal area in the WFDP at larger quadrat scales (Fig. 6). In contrast, the empirical skewness more closely tracked the simulations at YFDP. There is clear gradient of large-diameter tree density from east to west in the WFDP (Fig. S4) while large-diameter trees are more homogeneously distributed within the YFDP (Fig. S5). The inhomogeneous distribution of large-diameter trees within the WFDP maintains skewness of quadrat basal area at elevated values, even at larger quadrat sizes (Fig. 6). An important hypothesis that follows from this finding is that forest structural diversity responds to intermediate-scale environmental heterogeneity and disturbances, in the same way that patterns of species richness, and richness-ecosystem function relationships (specifically biomass and productivity) are hypothesized to [16]. Large mapped forest plots such as the YFDP and WFDP, with detailed within-plot environmental spatial covariates, will be required to test these hypotheses.

## Materials and Methods

### Study Area

**Figure 7:**
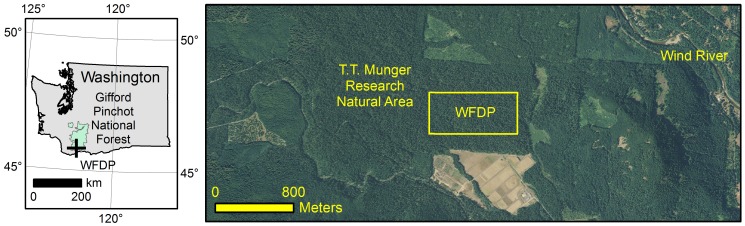
The Wind River Forest Dynamics Plot (WFDP) is located in the T.T. Munger Research Natural Area of the Gifford Pinchot National Forest (left, green) in the *Tsuga heterophylla* zone of western Washington, USA. The plot is located in a patch of late-successional forest. The area surrounding the Research Natural Area has had ongoing harvesting beginning in the late 19^th^ century.

**Figure 8:**
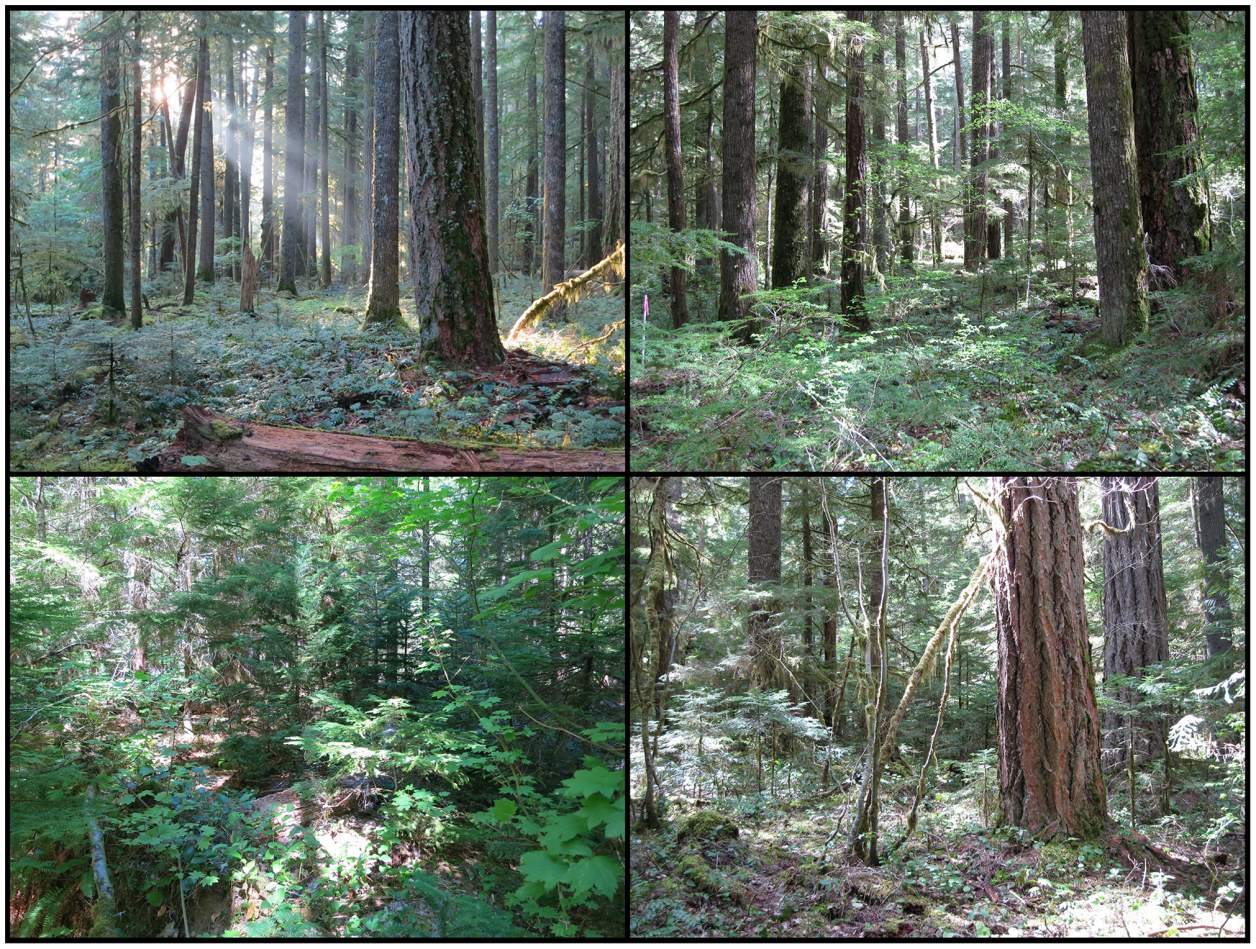
Structure and composition of the Wind River Forest Dynamics Plot (WFDP). Four images from different parts of the WFDP illustrate defining characteristics of the ecosystem. The forest is composed of an overstory of large-diameter trees with abundant but heterogeneous woody low shrub and herbaceous layers. Vegetation communities include *Tsuga heterophylla/Mahonia nervosa* (upper left image, August 19, 2012), and *Tsuga heterophylla/Mahonia nervosa/Gaultheria shallon* (upper right image, May 8, 2010). Regeneration by the shade-tolerant *Tsuga heterophylla* can be locally dense (lower right image, August 31, 2012). The most abundant species by stems, *Acer circinatum*, creates a seasonal sub-canopy layer (lower left image, May 8, 2010). All photos by J. A. Lutz.

The WFDP is located in the *Pseudotsuga/Tsuga* (Douglas-fir/western hemlock) forest in the T.T. Munger Research Natural Area of the Gifford Pinchot National Forest in western Washington State, USA. The plot is approximately oriented to the cardinal directions with dimensions of 800 m east to west and 320 m north to south (25.6 ha) centered at 45.8197° N, 121.9558° W (Fig. 7). Elevation ranges between 352.4 m and 384.7 m for a vertical relief of 32.3 m (Fig. S6). Soils are relatively deep tephra-derived Entic Vitrands overlaid on Quaternary olivine basalts [34]. The WFDP is comprised of vegetation types from the *Tsuga heterophylla* Zone [41], [42] including, from wet to dry, *Tsuga heterophylla/Athyrium filix-femina, Tsuga heterophylla/Tiarella trifoliata, Tsuga heterophylla/Polystichum munitum, Tsuga heterophylla/Mahonia nervosa/Polystichum munitum, Tsuga heterophylla/Vaccinium ovalifolium/Cornus canadensis, Tsuga heterophylla/Vaccinium ovalifolium/Gaultheria shallon, Tsuga heterophylla/Achlys triphylla, Tsuga heterophylla/Mahonia nervosa, Tsuga heterophylla/Mahonia nervosa/Gaultheria shallon, Tsuga heterophylla/Cornus nuttallii/Achlys triphylla,* and *Tsuga heterophylla/Pseudotsuga menziesii/Holodiscus discolor* ([42]; Fig. 8). Canopy emergents, primarily *Pseudotsuga menziesii* and *Tsuga heterophylla*, with minor contributions from *Pinus monticola*, *Abies procera*, and *Thuja plicata*, reach 60 m to 67 m in height. Tall shrubs, principally *Acer circinatum* and *Rhododendron macrophyllum*, constitute a distinct sub-canopy layer. Continuous patches of low shrubs (<1 m tall) and ferns cover much of the forest floor. Essentially all woody and herbaceous species are shade-adapted perennials [43]. Plant nomenclature follows Flora of North America [44].

The climate of the WFDP is a cool Mediterranean type, with cool moist winters and long dry summers. Between 1971 and 2000, the modeled mean temperature range at the WFDP was from 10.0°C to 26.8°C in July (16.8°C mean daily range) and −2.2°C to 4.2°C in January (6.4°C mean daily range); mean annual precipitation was 2,493 mm with 73% of precipitation falling in the winter months (November 1^st^ to March 31^st^), much of it as snow [45], [46]. Snow is intermittent, with mean annual peak accumulations of 50 cm snow water equivalent (SWE). Snow depth on April 1^st^ is generally 50 cm to 100 cm. The seasonality of precipitation can yield a seasonal drought, mediated in normal or wet years by soil water storage – an important aspect of plant moisture availability (calculation according to the methods of [47]; Fig. S7).

Fire is the primary stand-initiating disturbance in the western Cascade Range. Fire return intervals are on the order of several centuries, and fires burn at generally high severity. After fire, forest development is characterized by an initial cohort of shade-intolerant *Pseudotsuga menziesii* which can persist for up to a millennium [1]. Post establishment, the principal intermediate disturbance is wind, or wind in conjunction with snow [48]. There is no evidence of a past surface fire regime at the WFDP. Winter storms capable of killing multiple overstory trees have a frequency of once or twice per decade [49].

Insects are important agents of mortality, with *Dendroctonous pseudotsugae* (Douglas-fir bark beetle; affecting *Pseudotsuga menziesii*) and *Scolytus ventralis* (fir engraver beetle; affecting *Abies* spp.). *Dendroctonous ponderosae* (mountain pine beetle) affects *Pinus monticola* [50].

Pathogens include the structural root rots *Armillaria solidipes, Phellinus weirii*, and *Phaeolus schweinitzii*. The root rots spread through roots and root contacts at rates of approximately 30 cm per year, and hence tend to occur in patches [51]. *Armillaria solidipes* is a generalist pathogen and affects *Pseudotsuga*, *Abies*, *Acer*, and *Taxus*. *Phellinus weirii* affects *Pseudotsuga* and *Abies grandis*, and *Phaeolus schweinitzii*, a heart rot, tends to affect older *Pseudotsuga*, leading to structural failure. *Pinus monticola* is also affected by the introduced pathogen *Cronartium ribicola*, which has led to considerable mortality and reduced regeneration of this species since *Cronartium ribicola* reached the Gifford Pinchot National Forest in the 1930s. *Abies* spp. and *Tsuga heterophylla* are hosts to dwarf mistletoes: *Arceuthobium abietinum* on *Abies* spp., and *Arceuthobium tsugense* on *Tsuga heterophylla* [52]. These parasitic plants are distributed both by birds and by explosive discharge [53].

### Surveying

We established the WFDP following the same procedures used for the companion study at the Yosemite Forest Dynamics Plot [6], with the relevant methods also summarized here. We established a sampling grid using Total Stations with accuracies of 3–5 seconds of arc (Trimble S6, Nikon NPL-821, and Leica models Builder R200M Power and Builder 505). We set permanent markers on nominal 20 m centers, offset for tree boles or coarse woody debris. In addition to the sampling grid, we established two control points in open areas adjacent to the plot where good Global Positioning System (GPS) reception was possible. Two survey-grade GPS receivers (Trimble R6) were used to establish control to and across the plot. The GPS receivers collected data at 5 second intervals for 3 hours. The static GPS measurements were post-processed with Trimble Geomatics Office software (Trimble Inc., Sunnyvale, California) and grid locations calculated, with final grid point accuracies to the datum in the range of 0.10 m horizontally. We transformed the plot grid to Universal Transverse Mercator coordinates with Corpscon software (US Army Corps of Engineers). We augmented the ground survey with aerial LiDAR data acquired on 7 September 2011 by Watershed Sciences Inc., Corvallis, Oregon. Point density was 38.6 returns per m^2^ (ground return point density of 1.4 points per m^2^), with vertical and horizontal root mean square error of 0.03 m. Elevation of the 20 m grid locations was derived from the vendor-supplied LiDAR ground model. Grid elevations were not verified with Total Stations, but we have previously achieved 0.15 m vertical root mean square error with this method in similar forests [6].

### Field Sampling of Trees, Shrubs, and Snags

In the summers of 2010 and 2011 we tagged and mapped all live trees ≥1 cm at breast height, following the methods of Condit [54], with some alterations. We measured tree diameter at 1.37 m (instead of 1.30 m), and trees large enough to accept a nail were nailed at the point of measurement, both in keeping with research methods of the western United States. We measured tree locations from the surveyed grid points with a combination of hand-held lasers (Laser Technologies Impulse 200 LR), mirror compasses, and tapes. Tapes were laid south to north between adjacent grid points. We calculated the location of the tree centers from the horizontal and perpendicular references to the surveyed grid points and dbh with the assumptions of cylindrical boles and linear interpolation of elevation between adjacent grid points. All measurements were slope corrected. In addition to live trees, we tagged and mapped dead trees ≥10 cm dbh and ≥1.8 m in height. For each snag, we collected height, top diameter (with a laser), and snag decomposition class data (class 1 = least decayed, class 5 = most decayed). We mapped continuous patches of low shrubs and ferns ≥2 m^2^ relative to the 20 m sampling grid with a combination of tapes, mirror compasses, and lasers. For each shrub patch we recorded the shape of the patch as a polygon, as well as average and maximum shrub heights. Tree data were verified in 2012. This research was performed under a permit from the US Forest Service Pacific Northwest Research Station dated 2/9/2010.

### Quantifying Spatial Pattern

We analyzed spatial pattern identically to the methods of [6], which we summarize here. We quantified global spatial patterns with the univariate and bivariate forms of Ripley’s *K* function, using the square root (*L* function) transformation in all cases. For a given fully mapped pattern, an estimate of the *L(r)* function, the statistic 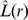, is based on the count of neighboring points occurring within a circle of radius *r* centered on the *i*th point, summed over all points in the pattern [40], [55]. The bivariate form 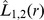 is a straightforward extension of the univariate case: it is the count of type 2 points occurring within a circle of radius *r* of the *i*th type 1 point, summed over all type 1 points in the pattern. We characterized patterns at interpoint distances from 0 m to 80 m (one quarter the minimum plot dimension) and used isotropic edge correction to account for points located closer than *r* to a plot edge [55]. Our study area included enough large-diameter trees to analyze spatial patterns of three tree species: *Pseudotsuga menziesii*, *Tsuga heterophylla* and *Thuja plicata*.

### Inferential Framework for Spatial Analyses

Univariate tree patterns were compared against a null distribution generated by a completely spatially random (CSR) process. Under CSR the location of each point in the pattern is completely independent of the locations of other points in the pattern. Positive values of 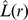 indicate spatial clustering (trees have more neighbors than expected under CSR) while negative values of 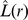 indicate spatial inhibition or uniformity (trees have fewer neighbors than expected under CSR).

Bivariate tree patterns were evaluated against the hypothesis of no interaction between the large-diameter and small-diameter subpopulations. We evaluated this hypothesis using the null model of population independence based on the guidelines of Goreaud & Pélissier [56]. Population independence is evaluated by holding the relative intratype spatial configuration constant (i.e., the relative tree locations within a diameter class are fixed) while subjecting the populations to random toroidal shifts. Under population independence significantly positive values of 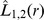 indicate a spatial attraction between the two types (e.g., originating from a parent-offspring relationship or facilitation) while significantly negative values indicate spatial repulsion between the two types (e.g., Janzen-Connell effects or competition). Large-diameter trees were ≥100 cm dbh; small-diameter trees were <100 cm dbh.

We used the 9 m radius neighborhood size estimated by Das et al. [8], [32] for Sierra Nevada mixed-conifer forests and tested the respective empirical patterns against the corresponding null models over 0 m≤*r*≤9 m using the goodness-of-fit test developed by Loosmore and Ford [57]. We set α = 0.05 and used *n* = 999 simulated patterns in each test. To control for multiple tests (*n* = 12) we used the Bonferroni correction, resulting in a threshold *P*-value of 0.004. Because we had no *a priori* hypotheses about tree patterns at spatial scales >9 m, we investigated patterns at larger scales in an exploratory framework by comparing the empirical 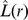 curves to the full distribution of 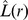 curves calculated for the simulated patterns. All analyses were implemented in the statistical program R version 2.14.1 [58]. Spatial analyses were conducted using the spatstat package version 1.25-1 [59].

### Simulation Experiment

We investigated the effect of global spatial pattern of large-diameter trees on heterogeneity of basal area with a simulation experiment. We simulated a pattern gradient spanning from strong spatial uniformity, to spatial randomness, to strong spatial aggregation (Fig. S1). Spatial randomness was simulated by shifting large tree locations by an independent random displacement using the rjitter function in spatstat. A gradient of increasing spatial uniformity of large tree locations was simulated using simple sequential inhibition with the SSI function in spatstat, with inhibition radii (i.e. the minimum allowable distance between points) of 10 m, 15 m, and 20 m. We simulated spatially aggregated patterns by first generating a realization of the Matern cluster process then randomly thinning the resulting pattern until the number of remaining points was equal to the number of large-diameter trees in the respective datasets. We generated a gradient of increasing spatial aggregation by setting the cluster radius of the Matern process to 20 m, 15 m, and 10 m, respectively. We set the intensity parameter for cluster centers (the kappa argument in the rMatClust function) to 0.00055 and set the mean number of points per cluster (the mu argument in the rMatClust function) to 5. We simulated n = 50 realizations of each of the seven pattern types.

In each simulation run new coordinates were generated for each tree ≥100 cm dbh while the locations of trees <100 cm dbh were held constant in their actual locations. After permuting large tree locations according to the respective spatial point process models, basal area for both large- and small-diameter trees in each quadrat was summed. We then evaluated the sensitivity of plot-wide heterogeneity of basal area to the global spatial pattern of large-diameter trees using the CV and skewness of the empirical quadrat basal area frequency distribution. We evaluated the effect of spatial scale of observation on basal area heterogeneity by conducting the analysis at four quadrat grains: 100 m^2^, 400 m^2^, 1600 m^2^, and 6400 m^2^ (c.f. [34]).

## Supporting Information

Figure S1Univariate spatial patterns of tree species that attain large diameters in the Wind River Forest Dynamics Plot at intertree distances up to 10 m. Solid black lines show the 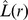 statistic for the actual patterns, where *r* is the intertree distance; thin gray lines show 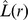 curves for 999 simulations of complete spatial randomness. Positive values of 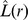 indicate spatial clumping and negative values of 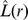 indicate spatial regularity.(TIF)

Figure S2Univariate spatial patterns for abundant small-diameter tree species in the Wind River Forest Dynamics Plot at intertree distances up to 10 m. Solid black lines show the 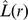 statistic for the actual patterns, where *r* is the intertree distance; thin gray lines show 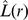 curves for 999 simulations of complete spatial randomness. Positive values of 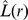 indicate spatial clumping and negative values of 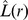 indicate spatial regularity.(TIF)

Figure S3Spatial interactions between large-diameter and small-diameter trees in the Wind River Forest Dynamics Plot at intertree distances up to 10 m. Solid black lines show the 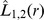 statistic for the actual pattern, where *r* is the intertree distance; thin gray lines show 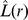 curves for 999 patterns simulated by synchronous random torodial shifts of large and small tree subpopulations. Positive values of 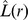 indicate spatial clumping and negative values of 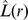 indicate spatial regularity.(TIF)

Figure S4 Actual spatial locations of large-diameter trees in the Wind River Forest Dynamics Plot in 2012 and example spatial patterns generated from the respective spatial point process models used in the simulation experiment.
(TIF)

Figure S5 Actual spatial locations of large-diameter trees in the Yosemite Forest Dynamics Plot in 2012.
(TIF)

Figure S6 Topography of the Wind River Forest Dynamics Plot. Topography derived from a LiDAR ground model at 1 m resolution (5 m contours; lighter colors represent higher elevations). Dots indicate corners of each 20 m×20 m quadrat of the 800 m×320 m plot. Elevation ranges from 352.4 m to 384.7 m for a vertical relief of 32.3 m. Drainages contain vernal streams.
(TIF)

Figure S7 Climatology and water balance of the Wind River Forest Dynamics Plot. The combination of temperature and precipitation (A) give rise to a mild summer drought in wet to normal years (B). Potential evapotranspiration (PET) exceeds available water supply from June through September, especially during dry years. Climate is represented by 1971–2000 climate normals from PRISM (2004).
(TIF)
