# Supplementary figures and images for: The Importance of Large-Diameter Trees to Forest Structural Heterogeneity
Source: PLoS One. 2013 Dec 20;8(12):e82784. doi: 10.1371/journal.pone.0082784 (PMC3869720; doi:10.1371/journal.pone.0082784)

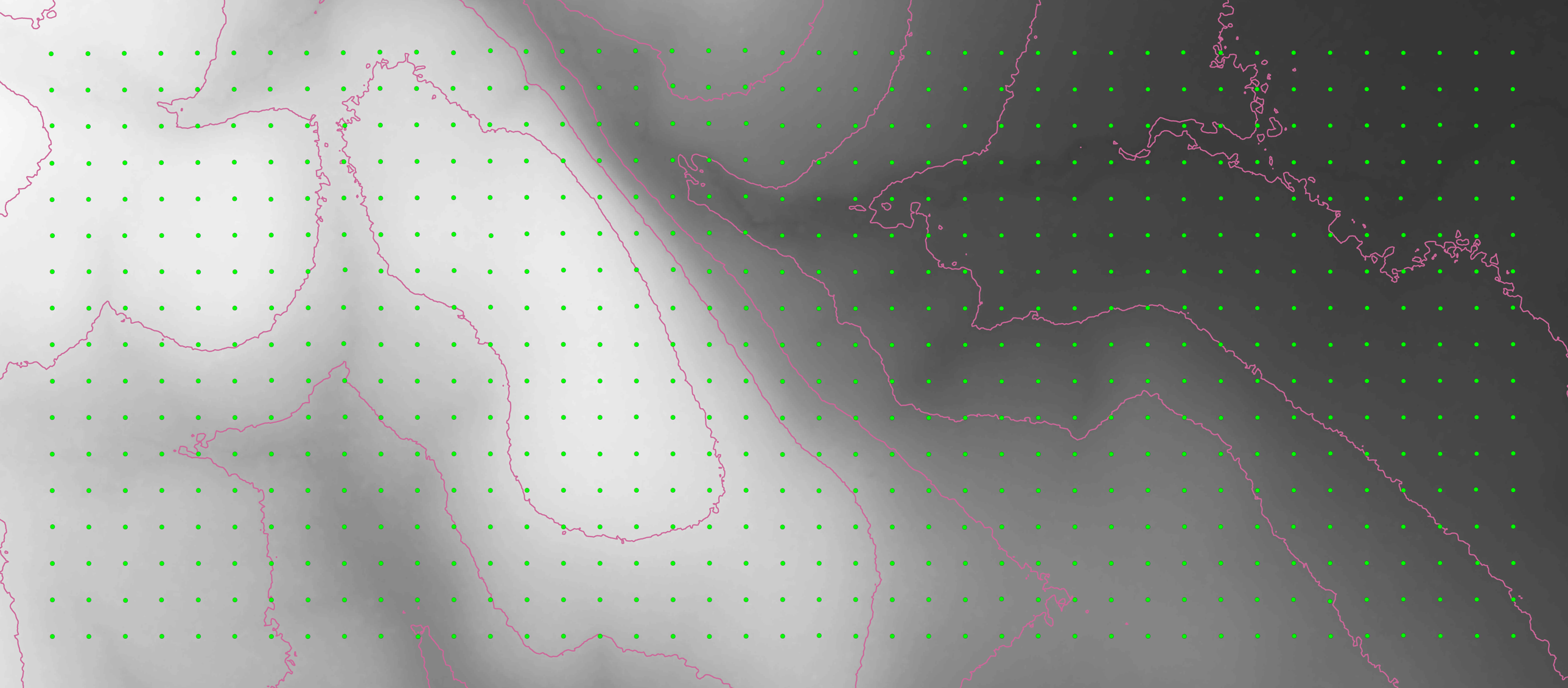

Supplement: Figure S6 — Topography of the Wind River Forest Dynamics Plot. Topography derived from a LiDAR ground model at 1 m resolution (5 m contours; lighter colors represent higher elevations). Dots indicate corners of each 20 m×20 m quadrat of the 800 m×320 m plot. Elevation ranges from 352.4 m to 384.7 m for a vertical relief of 32.3 m. Drainages contain vernal streams. (TIF) [file pone.0082784.s006.tif]
